# HDEC: A Heterogeneous Dynamic Ensemble Classifier for Binary Datasets

**DOI:** 10.1155/2020/8826914

**Published:** 2020-12-14

**Authors:** Nasrin Ostvar, Amir Masoud Eftekhari Moghadam

**Affiliations:** Faculty of Computer and Information Technology, Qazvin Branch, Islamic Azad University, Qazvin, Iran

## Abstract

In recent years, ensemble classification methods have been widely investigated in both industry and literature in the field of machine learning and artificial intelligence. The main advantage of this approach is to benefit from a set of classifiers instead of using a single classifier with the aim of improving the prediction performance, such as accuracy. Selecting the base classifiers and the method for combining them are the most challenging issues in the ensemble classifiers. In this paper, we propose a heterogeneous dynamic ensemble classifier (HDEC) which uses multiple classification algorithms. The main advantage of using heterogeneous algorithms is increasing the diversity among the base classifiers as it is a key point for an ensemble system to be successful. In this method, we first train many classifiers with the original data. Then, they are separated based on their strength in recognizing either positive or negative instances. For doing this, we consider the true positive rate and true negative rate, respectively. In the next step, the classifiers are categorized into two groups according to their efficiency in the mentioned measures. Finally, the outputs of the two groups are compared with each other to generate the final prediction. For evaluating the proposed approach, it has been applied to 12 datasets from the UCI and LIBSVM repositories and calculated two popular prediction performance metrics, including accuracy and geometric mean. The experimental results show the superiority of the proposed approach in comparison to other state-of-the-art methods.

## 1. Introduction

Classification is a type of supervised learning which is aimed at predicting the class of given data samples. There are many classification algorithms in the literature including decision trees, support vector machines [1], neural networks [2, 3], Bayesian networks [4], and fuzzy classifiers [5–12]. However, according to the “No Free Launch” theorem, there is no universally superior classification algorithm that can outperform other algorithms for all datasets [13]. A good solution to deal with this problem is the development and use of ensemble classification algorithms [14]. In ensemble learning, instead of using a single classification algorithm, a set of algorithms are considered and the final output is generated by combining the output of each classifier. Indeed, the main goal of an ensemble classifier is to benefit from the advantages of multiple classifiers and combine their outputs such that the quality of the final output improves [15]. The individual classifiers contained in an ensemble system are called base classifiers.

The main challenges with ensemble classifiers are (1) to select the base classifiers and (2) to combine the output of the base classifiers [16]. An essential key for designing a successful ensemble is to ensure that the base classifiers are sufficiently diverse [17–19]. Two classifiers are diverse if their outputs do not correlate with each other [20]. There are different strategies for the construction of an ensemble system. In homogeneous ensembles, the base classifiers are from the same family and the diversity among the base classifiers is achieved by training them with different samples of the training dataset [21].

Conversely, in heterogeneous ensembles, different classification algorithms are considered for the base classifiers; therefore, diversity is achieved by using different algorithms [22]. Bagging [23] and Boosting [24] are the most commonly used homogeneous classifiers in the literature. In Bagging, each base classifier is trained with a bootstrapped replica of the training dataset and the final decision is generated by applying a majority voting on the decision of each base classifier. It is worth noting that other combination methods can also be found in the literature such as weighted voting and plurality voting [25]. On the other hand, the Boosting constructs the base classifiers in an iterative fashion, each compensating the weakness of its predecessors [26]. The constructed base classifiers are finally integrated using the weighted voting approach.

**Figure 1:**
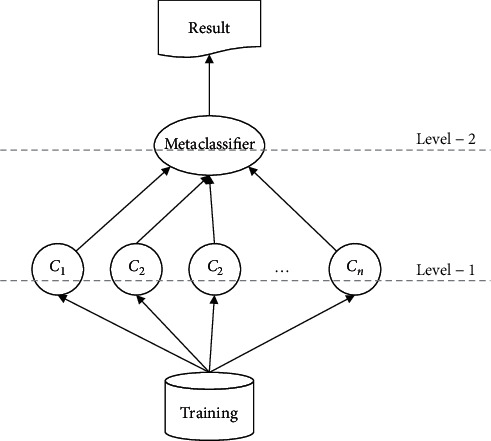
The Stack Generalization approach. In the first level, some heterogeneous classifiers are trained with the whole dataset and in the second level the output of the classifiers is combined through the metaclassifier.

Focusing on increasing diversity, several papers have proposed heterogeneous ensemble classifiers [16, 27–29]. Stack Generalization (briefly, Stacking) is one the most successful heterogeneous ensembles proposed in 1992 [30]. As shown in Figure 1, it uses a two-layer schema for training the model. In the first layer, the base classifiers are trained with different classification algorithms on the original dataset. For example, in the first layer, one classifier is trained with a Naïve Bayes algorithm while the other classifier uses a decision tree algorithm for training on the dataset. After training the base classifiers in the first layer, the output of them for the same original dataset is used as a new dataset to train the metaclassifier in the second layer. It means that the output of each classifier in the first level is considered as the inputs of the metaclassifier in the second level. It is worth noting that selecting the type of the base classifiers in the first level is an important issue in the Stacking, because it could affect the prediction performance of the Stacking. As mentioned before, an ideal subset of the base classifiers are those that are diverse and accurate.

The prediction performance of the Stacking is tightly dependent on the accuracy and diversity of the base classifiers in the first layer [22, 28, 31]. In this paper, we propose a novel approach for the smart selection of the base classifiers in order to improve the prediction performance of the final model. We categorized the base classifiers into two groups such that the classifiers within the first group can efficiently recognize the positive samples while the second group contains the classifiers suitable for the negative samples.

In this paper, we propose a heterogeneous dynamic ensemble classifier (HDEC) which uses multiple classification algorithms and is aimed at selecting the best classifiers for positive and negative instances. We categorize the classifiers using the true positive rate and true negative rate values to specify which classifiers are more accurate for recognizing the positive and negative instances. Then, for an unseen instance, the weight of each category is compared to generate the final output. Our major contributions are the following:

1. We design a novel ensemble classifier which uses heterogeneous classification algorithms in order to increase the diversity between the base classifiers and benefits from the advantages of different algorithms.
2. HDEC dynamically selects the base classifiers that should participate in the final decision-making process. In other words, depending on the test instances, it is possible that different classifiers are selected to generate the final output.
3. HDEC considers both TPR and TNR for detecting the suitable classifiers in recognizing the positive and negative instances, respectively. Indeed, unlike the existing methods, it does not rely on just the accuracy of classifiers and with using the TPR and TNR concepts it generates a more powerful ensemble classifier.

In order to evaluate the proposed approach, we have applied it on 12 datasets from the UCI and LIBSVM repositories. We use two popular performance measures including accuracy and geometric mean. The obtained results show the superiority of the HDEC in comparison to individual classifiers and state-of-the-art ensemble approaches.

The rest of this paper is organized as follows.  Section 2 contains a brief review of previous related works. In Section 3, we present our approach, while Section 4 demonstrates the experimental results and shows the comparisons with different approaches. Finally, in Section 5, we conclude this study along with some future research directions.

## 2. Related Work

Several papers in the literature have investigated the usage of the multiple classification algorithms as the base learners of the ensemble classifiers [16, 27, 28, 32–35]. There are two general approaches for selecting the base classifiers of an ensemble classifier: static approaches and dynamic approaches. In static approaches, the base classifiers that should participate in the final decision-making process are selected during the training the ensemble classifier. Mendialdua et al. [36] proposed an approach to improve the accuracy of the Stack Generalization. The authors argued that although using multiple classification algorithms increases the diversity among the base classifiers, not all of them are suitable to be combined for generating the final output. As a result, they appended an extra step for the Stack Generalization to prune similar classifiers by applying the Estimation of the Bayesian Network Algorithm (EBNA) on the base classifiers. Coelho and Nascimento [27] used five different learning algorithms for the base classifiers of the bagging approach. They mapped the combination of the base classifiers to the chromosomes and by applying the genetic algorithm they find the best combination of the base classifiers that should be aggregated for generating the final output. Kadkhodaei and Eftekhari [37] designed a genetic algorithm to find the most diverse set of the base classifiers to improve the accuracy of the Stacking. A heterogeneous boosting-based classifier is proposed in [38]. The authors have considered a set of distinct learning algorithms and generated heterogeneous classifiers at each iteration of the boosting. In order to increase the diversity among the base classifiers, a pruning step eliminates similar base classifiers. Finally, the remaining classifiers are combined by applying the weighted voting method.

In contrast, in dynamic ensemble classifiers (DES), the decision of which classifiers should be combined for generating the final output is postponed until generalization phase [39–45]. In other words, there will be not a fixed subset of classifiers which applies to any test instance. A dynamic ensemble classifier for credit scoring has been proposed in [46]. In this method, initially, the classifiers are selected based on their accuracy and relative costs of Type I and Type II errors for a validation set. Then, the selected classifiers are combined based on the classification results by using the soft probability. The authors have applied their approach to the four credit datasets from the UCI repository and reported the superiority of their algorithm in terms of the accuracy, area under ROC (receiver operating characteristic) curve (AUC), H-measure, and Partial Gini index (PG) in comparison to other state-of-the-art approaches. The authors of [47] argued that most misclassifications occur in areas near classes boundaries of a dataset. They called this area Region of Competence (RoC) and proposed an approach (FIRE-DES) to dynamically select the best base classifiers which are powerful in classifying the instance which falls into the RoC. They have applied their approach on 40 datasets and showed the validity of the FIRE-DES against other approaches.

## 3. HDEC: A Heterogeneous Dynamic Ensemble Classifier

In this section, we describe our proposed approach. Our main objective is to design a heterogeneous ensemble classifier such that the prediction performance improves.

**Figure 2:**
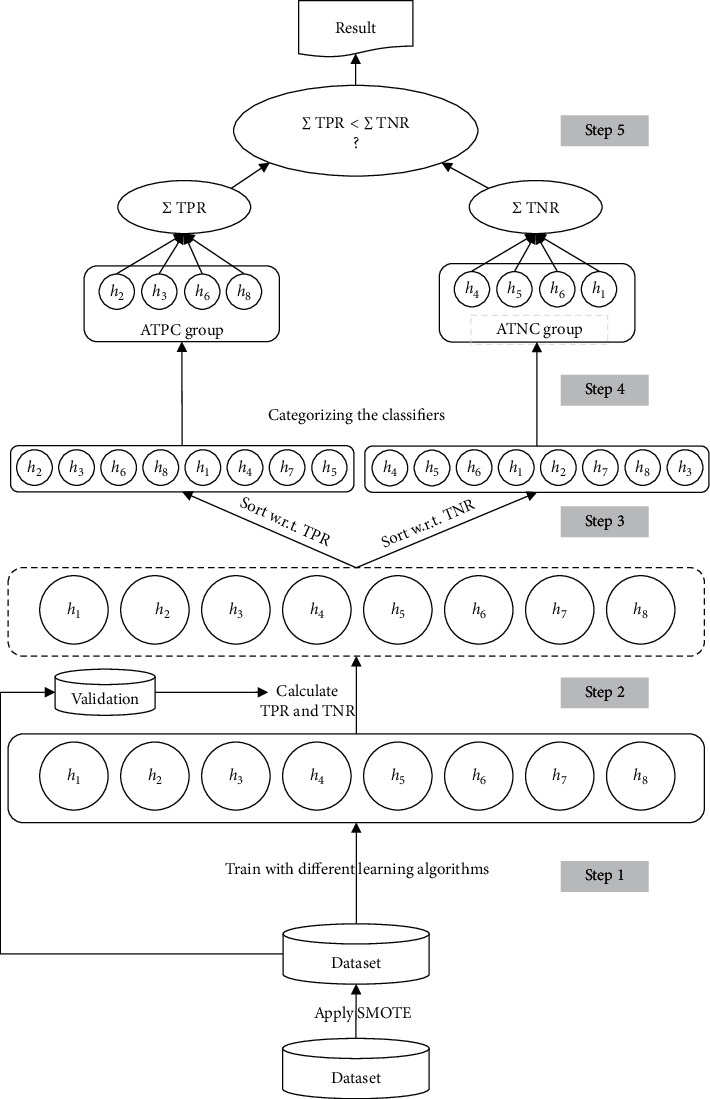
The general schema of the HDEC. Each *h*_*i*_ is a classifier trained by the whole dataset. The generated classifiers are split into two categories based on the TPR and TNR values. For classifying an unlabeled instance, the sum of the TPR and TNR in each category is calculated and the final output is the argmax between these two values.

**Figure 3:**
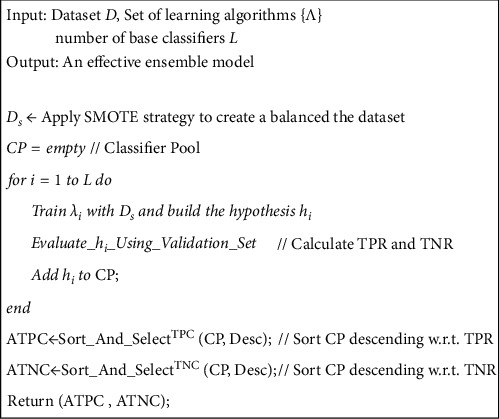
The pseudocode for training phase of HDEC.

**Figure 4:**
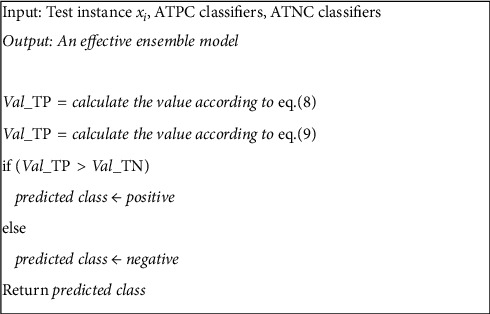
The pseudocode for generalization phase of HDEC.

Figure 2 depicts the general schema of the HDEC. The pseudocode of the training and generalization phase is also shown in Figures 3 and 4. In a nutshell, we use multiple learning algorithms and train many classifiers with them in order to separate the classifiers which are accurate in recognizing the positive samples and those that are efficient in recognizing the negative samples. The rationale behind separating the classifiers is that some classifiers which are efficient in recognizing the positive samples may not be suitable for classifying the negative samples. This is true for efficient classifiers in recognizing the negative samples. Afterward, we make two groups and for an unlabeled instance we dynamically specify which group is more confident for generating the final prediction of the instance.

As we can see in Figure 2, the proposed approach consists of five steps. Furthermore, a preprocessing step is required to make a balance dataset from the input instances. As mentioned before, the main idea behind the proposed approach is to use both true positive rate and true negative rate simultaneously for identifying which classifiers can better recognize the instances. In other words, the degree of trust in each classifier is determined by the obtained values of TPR and TNR.

Let us consider *𝒟*={(*x*_1_, *y*_1_), (*x*_2_, *y*_2_),…, (*x*_*N*_, *y*_*N*_)} is the dataset where each (*x*_*i*_, *y*_*i*_) represents a training sample such that *x*_*i*_ ∈ *ℝ*^*d*^ , *y*_*i*_ ∈ {+, −}, and *N* is the number of total samples. In the following, we elaborate on the steps of the proposed method. Since we use multiple learning algorithms, we can expect that a diverse set of classifiers are formed.

### 3.1. Generating Many Classifiers

Our proposed approach commences with considering many learning algorithms and training them by using the original dataset. Suppose that we consider *L* distinct learning algorithms. As a result, *L* classifiers will be generated:

(1) $$\begin{array}{c} H=\left\{h_{1},h_{2},\ldots ,h_{L}\right\}. \end{array}$$

In other words, the output of this step is a pool of *L* heterogeneous and diverse classifiers. Note that in our proposed method the dataset should have an equal number of positive and negative instances, i.e., a balance dataset. Thus, we have used the SMOTE technique [48] to convert the imbalanced dataset into the balanced dataset.

### 3.2. Calculating the TPR and TNR

In order to specify which classifiers are efficient for classifying either positive or negative samples, we consider a validation set. Each classifier generates the predictions for the instances within the validation set.

Then, we evaluate the performance of the classifier for positive and negative samples by calculating the TPR and TNR according to (2) and (3), respectively. TPR or recall [49] is defined as the ratio of the positive samples (TP) that are correctly classified to the total number of positive samples in the validation set (P).

The more the positive samples are classified correctly, the higher the TPR value is. Accordingly, TNR or specificity represents the ratio of the number of negative samples correctly classified (TN) to the total number of negative samples (*N*) [49]:

(2) $$\begin{array}{c} \text{TPR}=\frac{\text{TP}}{P}, \end{array}$$

(3) $$\begin{array}{c} \text{TNR}=\frac{\text{TN}}{N}. \end{array}$$

### 3.3. Sorting the Classifiers with respect to the TPR and TNR

After calculating the TPR and TNR for the generated classifiers, we sort them with respect to both TPR and TNR in descending order. The reason for sorting the classifiers is that we want to specify which of them are more efficient for positive samples and which of them are more efficient for the negative samples. We have

(4) $$\begin{array}{c} H=\left\{h_{1},h_{2},\ldots ,h_{L}\right\}\overset{\text{sort w.r.t. TPR}}{⟶}H^{\text{TPR}}:\;\left\{h_{1}^{\text{TPR}},h_{2}^{\text{TPR}},\;\ldots ,\;h_{L}^{\text{TPR}}\right\}, \end{array}$$

such that TPR(*h*_*i*_^TPR^) > TPR(*h*_*j*_^TPR^),  *i* < *j*

(5) $$\begin{array}{c} H=\left\{h_{1},h_{2},\ldots ,h_{L}\right\}\overset{\text{sort w.r.t. TNR}}{⟶}H^{\text{TNR}}:\;\left\{h_{1}^{\text{TNR}},h_{2}^{\text{TNR}},\;\ldots ,\;h_{L}^{\text{TNR}}\right\}, \end{array}$$

such that TNR(*h*_*i*_^TNR^) > TNR(*h*_*j*_^TNR^),  *i* < *j*

### 3.4. Categorizing the Classifiers into Two Groups

After sorting the classifiers in the previous step, we select the top *T* of the sorted classifiers to make two groups:

ATPC (Accurate True Positive Classifiers): this group contains the classifiers which accurately recognize the positive samples. We have

(6) $$\begin{array}{c} H^{\text{TPR}}:\;\left\{h_{1}^{\text{TPR}},h_{2}^{\text{TPR}},\;\ldots ,\;h_{L}^{\text{TPR}}\right\}\overset{\text{top }T}{⟶}H^{\text{TPR}}:\;\left\{h_{1}^{\text{TPR}},h_{2}^{\text{TPR}},\;\ldots ,\;h_{T}^{\text{TPR}}\right\}. \end{array}$$

ATNC (Accurate True Negative Classifiers): the classifiers within this group are efficient in recognizing the negative samples:

(7) $$\begin{array}{c} H^{\text{TNR}}:\;\left\{h_{1}^{\text{TNR}},h_{2}^{\text{TNR}},\;\ldots ,\;h_{L}^{\text{TNR}}\right\}\overset{\text{top }T}{⟶}H^{\text{TNR}}:\;\left\{h_{1}^{\text{TNR}},h_{2}^{\text{TNR}},\;\ldots ,\;h_{T}^{\text{TNR}}\right\}. \end{array}$$

### 3.5. Combining the Classifiers

Once the classifiers are divided into two groups, there should be a strategy to combine the output of the classifiers. For an unlabeled sample, the classifiers within each group generate their prediction. For the ATPC group, we only consider the positive prediction and for the ATNC group we only consider the negative predictions (according to (8) and (9)). This is due to the fact that the classifiers in the ATPC group are just efficient in recognizing the positive samples and generating a negative prediction does not matter much. In contrast, if a classifier in the ATNC group generates a positive prediction for an unlabeled instance, we ignore it. Afterward, we compare the sum of TPR and TNR for the ATPC and ATNC groups, respectively. The final prediction is the class that generates the highest value:

(8) $$\begin{array}{c} {\text{predict}}_{\text{ATPC}}=\overset{N_{\text{ATPC}}}{\underset{i=1}{\sum }}\text{TPR}\left(h_{i}^{\text{TPR}}\right)\times I\left(\text{predict}\left(x_{i}\right)=\text{positive}\right), \end{array}$$

(9) $$\begin{array}{c} {\text{predict}}_{\text{ATNC}}=\overset{N_{\text{ATNC}}}{\underset{i=1}{\sum }}\text{TNR}\left(h_{i}^{\text{TNR}}\right)\times I\left(\text{predict}\left(x_{i}\right)=\text{negative}\right), \end{array}$$

where *I*(·) is the identity function such that its output is one if the input parameter is true; otherwise it is zero. In (8), TPR(*h*_*i*_^TPR^) is the true positive rate value for the base classifier *h*_*i*_. This value is calculated in the second step of the proposed algorithm as described in Section 3.2. The expression *I*(predict(*x*_*i*_)=positive) is for ignoring the negative prediction by the classifiers in the ATPC group. In other words, if a classifier in the ATPC group makes a negative prediction for an unlabeled instance *x*_*i*_, the value of *I*(predict(*x*_*i*_)=positive) will be zero, because the input parameter is false. Consequently, the TPR value is ignored as it will be multiplied by the zero.

According to the above description, it makes sense that HDEC dynamically specifies which category is more confident for generating the final output for an unlabeled instance.

## 4. Experimental Study

In this section, we describe the experimental details and the analysis carried out to show the performance of the proposed method against state-of-the-art approaches.

### 4.1. Dataset

For evaluating the proposed method, 12 standard datasets from the UCI [50] and LIBSVM [51] repositories are selected.  Table 1 shows the characteristics of the used datasets. The last column indicates the ratio of the majority class to the minority class. As it can be seen, the selected datasets belong to different categories and cover a wide range of problems (from 155 to 1284 instances, from two to 60 features, and from a variety of imbalance ratios). Since we categorize the classifiers into two groups, our approach is only applicable to the binary datasets. To carry out the experiments, we have used a 10-fold cross-validation strategy. That is, each dataset is divided into ten folds and we use nine folds for training the classifiers and the remaining fold as the test data. We repeat this process ten times and finally report the average value of the performance measures.

### 4.2. Base Classifiers

All of the experiments have been carried out by using the Weka package which is one of the most popular machine learning libraries.  Table 2 presents the classification algorithms used as the base classifiers of the proposed method. As can be seen, they belong to the different families of the Weka classifiers.

### 4.3. Performance Measurements

To reach a reliable result on the proposed approach, we have used two popular performance indicator measures including accuracy and geometric mean. The accuracy is calculated from dividing the number of instances correctly classified by the total number of instances. It is formulated by [52]

(10) $$\begin{array}{c} \text{accuracy}=\frac{\text{TP}+\text{TN}}{N}\times 100, \end{array}$$

where TP is the number of positive instances that are correctly recognized and TN is the number of negative instances that are correctly classified.

However, the accuracy alone does not express which class has been better classified. Regarding this issue, we use geometric mean measure which considers the strength of a classifier in both positive and negative classes [52]:

(11) $$\begin{array}{c} \text{GM}=\sqrt{\text{TPR}\times \text{TNR}}=\sqrt{\frac{\text{TP}}{\text{TP}+\text{FN}}\times \frac{\text{TN}}{\text{TN}+\text{FP}}}. \end{array}$$

### 4.4. Method

With the aim of evaluating the proposed approach, we have compared HDEC against different classifiers including (a) single classifiers presented in Table 2, (b) three state-of-the-art ensemble classifiers investigated in the literature including Bagging, Boosting, and Stacking, and (c) the approach introduced in [36].

### 4.5. Obtained Results

Table 3 shows the best average accuracy and geometric mean for the individual classifiers presented in Table 2 along with the corresponding classification algorithm. The standard deviations of the accuracies and geometric means of 10 simulations are also shown using the “±” sign corresponding to each percentage.

As it is clear, there is not a unique classification algorithm that is always superior to others. For example, while the decision tree generates the best accuracy for the BRC and COL datasets, the SVM has the highest accuracy for the DBT and HPT datasets.

Tables 4 and 5 show the values of the accuracy and geometric mean of different ensemble approaches, respectively. For doing this, we have set each classification algorithm in Table 2 as the base learner of Bagging and Boosting.

**Figure 5:**
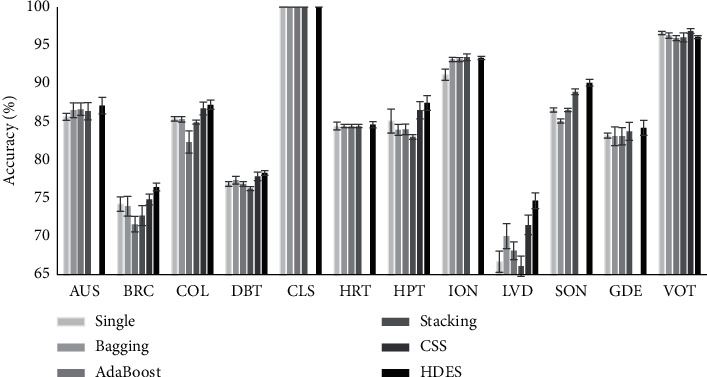
The comparison of the accuracy of the HDEC and other approaches.

**Figure 6:**
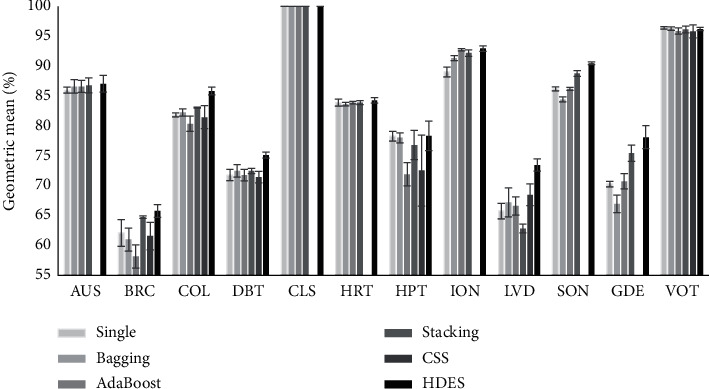
The comparison of the geometric mean of the HDEC and other approaches.

For Stack Generalization, we have set the classification algorithm as its metaclassifiers. The hyphen for the CSS column indicates that the authors of [36] did not applied their approach for the respective dataset. The best results are shown in bold fonts. The results are shown graphically in Figures 5 and 6. As can be seen, HDEC outperforms the other ensemble approaches in 10 out of 12 datasets in terms of the accuracy and 11 out of 12 datasets in terms of geometric mean. In other words, both accuracy and geometric mean have been improved as a result of applying the HDEC method.

We can also see that the geometric mean has been more improved than the accuracy. This is as a result of smart selection of the base classifiers for classifying the unseen instances. The positive instances are more likely to be classified correctly with the classifiers which are efficient in classifying the positive instances, i.e., ATPC groups. Accordingly, the same is true for negative instances. This causes the fact that both TPR and TNR values improve simultaneously and consequently the geometric mean value increases.

Table 6 shows the classifiers in each ATPC and ATNC group. It is observed that there are different classification algorithms which are suitable for classifying the positive and negative instances. For example, while *KStar* algorithm has a good strength for classifying the positive instances of the LVD dataset, it is suitable for neither classifying the positive instances nor classifying negative instances. It should be taken into account that a classification algorithm may have a good strength for classifying both positive and negative instances. As an exemplification, we can see that the Naïve Bayes classifier exists in both ATPC and ATNC groups which means that it can classify both positive and negative instances efficiently.

## 5. Conclusion

The aim of this study is to design a heterogeneous ensemble classifier in order to achieve higher prediction performance. For this purpose, we consider multiple classification algorithms and train each of them with the training dataset. Then, we separate the classifiers which are accurate in recognizing the positive samples and those that are efficient in recognizing the negative samples. This is done by calculating the TPR and TNR values of each classifier for the validation set. Afterwards, we select the classifiers which have the higher value for the TPR and TNR to form two groups ATPCS and ATNC classifiers, respectively. We then combine the output of the classifiers in each group to generate the final output.

In order to evaluate the prediction performance of the HDEC, we have applied it on 12 standard datasets from the UCI repository and compare our proposed method against three state-of-the-art ensemble approaches including Bagging, Boosting, and Stack Generalization. The obtained results show the superiority of HDEC in terms of the accuracy and geometric mean values.

It is worth noting that although HDEC shows better performance in many datasets, it is not our purpose to express that HDEC is always better than other ensemble approaches for any dataset. As we stated before, according to “No Free Lunch” theorem, there is not a unique classifier which always shows best performance for all datasets. The proposed approach needs a balanced dataset and for this purpose we included an extra preprocessing step (using SMOTE) to convert imbalanced datasets to the balanced datasets.

For future work, we plan to extend the proposed algorithm such that it does not need the balancing step and can handle the imbalanced datasets directly. Furthermore, it is suggested to develop an adoption model to investigate the usage of more classification algorithms as the base classifiers of the HDEC. We also plan to extend our work to be capable of classifying the multiclass datasets.

## Figures and Tables

**Table 1:** The characteristics of the used datasets.

Dataset	No. of samples	No. of features	Min/Maj
Australian	690	14	307/383
Breast-cancer	286	9	85/201
Colic	368	22	136/232
Diabetes	768	8	268/500
Four-class	862	2	307/555
Heart	270	13	120/150
Hepatitis	155	19	70/85
Ionosphere	351	34	126/225
Liver-disorders	345	6	145/200
Sonar	208	60	97/111
SVMguide	1284	21	296/947
Vote	435	16	168/267

**Table 2:** The classification algorithms used for the base classifiers.

Type	Base classifier	Notation
Bayes	Naïve Bayes	NB
	Bayesian network	BN

Lazy	KStar	K^*∗*^
	IBk	KNN

Tree	Decision tree	DT
	RepTree	RT
	Decision stump	DS

Rule‏Base
	OneR	1R
	Decision table	DsT

Functions
	SVM	SVM

**Table 3:** The best result of individual classifiers.

Dataset	Accuracy		Geometric mean	
	Type	Value	Type	Value
AUS	RT	85.65 ± 0.46	1R	85.98 ± 0.51
BRC	DT	74.23 ± 0.96	DS	62.11 ± 2.24
COL	DT	85.38 ± 0.29	DT	81.85 ± 0.34
DBT	SVM	76.86 ± 0.31	BN	71.81 ± 0.94
CLS	KNN	100	KNN	100
HRT	SVM	84.44 ± 0.51	SVM	83.90 ± 0.57
HPT	SVM	85.10 ± 1.57	NB	78.28 ± 0.83
ION	RT	91.16 ± 0.73	DT	89.02 ± 0.81
LVD	K^*∗*^	66.67 ± 1.39	K^∗^	65.76 ± 1.30
SON	KNN	86.54 ± 0.27	KNN	86.20 ± 0.33
GDE	DsT	83.19 ± 0.33	DT	70.27 ± 0.44
VOT	DT	96.64 ± 0.21	DT	96.39 ± 0.21

**Table 4:** The best accuracy of the ensemble approaches.

Dataset	Best-bagging		Best-AdaBoost		Best-stacking		CSS [36]	HDEC
	Type	Acc	Type	Acc	Type (meta)	Acc		
AUS	DT	86.52 ± 0.97	DS	86.67 ± 0.82	BN	86.38 ± 1.12	—	**87.12** ± 1.09
BRC	BN	73.95 ± 1.30	DS	71.61 ± 1.02	BN	72.73 ± 1.31	74.83 ± 0.70	**76.47** ± **0.52**
COL	RT	85.33 ± 0.36	DS	82.36 ± 1.46	BN	84.96 ± 0.26	86.74 ± 0.84	**87.23** ± 0.60
DBT	BN	77.36 ± 0.50	BN	76.86 ± 0.31	NB	76.26 ± 0.25	77.86 ± 0.56	**78.29** ± 0.31
CLS	KNN	100	KNN	100	All– (K^*∗*^, DsT)	100	—	**100**
HRT	NB	84.44 ± 0.21	SVM	84.44 ± 0.21	RT	84.44 ± 0.21	—	**84.63** ± 0.41
HPT	NB	83.94 ± 0.73	NB	84.00 ± 0.69	BN	83.01 ± 0.30	86.52 ± 1.12	**87.47** ± 0.97
ION	RT	93.16 ± 0.28	NB	93.15 ± 0.27	BN	**93.45** ± 0.44	—	93.36 ± 0.21
LVD	DT	70.03 ± 1.63	DT	68.12 ± 1.15	NB	66.09 ± 1.32	71.51 ± 1.27	**74.67** ± **1.02**
SON	K^*∗*^	85.10 ± 0.27	KNN	86.54 ± 0.22	RT	88.94 ± 0.37	—	**90.12** ± 0.44
GDE	DT	83.11 ± 1.24	DT	83.11 ± 1.13	SVM	83.75 ± 1.18	—	**84.21** ± 0.97
VOT	DT	96.30 ± 0.36	NB	95.95 ± 0.33	DS	96.07 ± 0.59	**96.90** ± 0.31	96.09 ± 0.21

**Table 5:** The best geometric mean of the ensemble approaches.

Dataset	Best-bagging		Best-AdaBoost		Best-stacking		CSS [36]	HDEC
	Type	Acc.	Type	Acc.	Type (meta)	Acc.		
AUS	DT	86.60 ± 1.13	DS	86.60 ± 1.01	BN	86.78 ± 1.27	—	**87.05** ± 1.41
BRC	BN	61.00 ± 1.92	DS	58.16 ± 1.95	BN	64.74 ± 0.20	61.57 ± 2.31	**65.77** ± 1.06
COL	RT	82.25 ± 0.59	DS	80.35 ± 1.27	BN	83.06 ± 0.09	81.47 ± 1.93	**85.85** ± 0.64
DBT	BN	72.48 ± 1.05	BN	71.78 ± 0.95	NB	72.46 ± 0.44	71.44 ± 0.96	**75.11** ± 0.51
CLS	KNN	100	KNN	100	All– (K^*∗*^, DsT)	100	—	**100**
HRT	NB	83.65 ± 0.29	SVM	83.90 ± 0.22	DS	83.90 ± 0.32	—	**84.29** ± 0.46
HPT	NB	78.01 ± 0.84	NB	71.92 ± 1.93	BN	76.81 ± 2.46	72.53 ± 5.93	**78.35** ± 2.46
ION	RT	91.34 ± 0.42	NB	92.73 ± 0.19	BN	92.18 ± 0.55	—	**92.94** ± 0.46
LVD	DT	67.23 ± 2.43	DT	66.61 ± 1.53	NB	62.83 ± 0.73	68.48 ± 1.82	**73.46** ± 1.02
SON	K^*∗*^	84.43 ± 0.42	KNN	86.20 ± 0.24	RT	88.77 ± 0.49	—	**90.48** ± 0.25
GDE	BN	66.97 ± 1.48	RT	70.74 ± 1.27	NB	75.44 ± 1.37	—	**78.12** ± 1.91
VOT	DT	**96.27** ± 0.31	NB	95.88 ± 0.52	DS	96.11 ± 0.56	95.80 ± 1.08	96.21 ± 0.29

**Table 6:** The selected classifiers of ATPC and ATNC groups.

Dataset	True positive classifiers	True negative classifiers
AUS	*NaiveBayes*, *BayesNet*, *KStar*, and *J48*	*OneR*, *DecisionStump*, *SMO*, and *DecisionTable*
BRC	*REPTree*, *J48*, *DecisionTable*, and *IBk*	*BayesNet*, *NaiveBayes*, *IBk*, and *DecisionTable*
COL	*DecisionTable*, *J48*, *REPTree*, and *SMO*	*OneR*, *DecisionStump*, *NaiveBayes*, and *SMO*
DBT	*SMO*, *OneR*, *DecisionTable*, and *NaiveBayes*	*BayesNet*, *REPTree*, *NaiveBayes*, and *J48*
CLS	*IBk*, *KStar*, *J48*, and *REPTree*	*IBk*, *KStar*, *J48*, and *DecisionStump*
HRT	*DecisionTable*, *SMO*, *NaiveBayes*, and *BayesNet*	*BayesNet*, *SMO*, *NaiveBayes*, and *DecisionTable*
HPT	*BayesNet*, *NaiveBayes*, *DecisionTable*, and *SMO*	*DecisionStump*, *REPTree*, *OneR*, and *SMO*
ION	*NaiveBayes*, *J48*, *REPTree*, and *BayesNet*	*DecisionStump*, *KStar*, *SMO*, and *IBk*
LVD	*NaiveBayes*, *KStar*, *IBk*, and *J48*	*SMO*, *DecisionTable*, *REPTree*, and *BayesNet*
SON	*KStar*, *IBk*, *DecisionTable*, and *BayesNet*	*IBk*, *NaiveBayes*, *KStar*, and *SMO*
GDE	*SMO*, *DecisionTable*, *OneR*, and *REPTree*	*BayesNet*, *J48*, *REPTree*, and *DecisionTable*
VOT	*J48*, *DecisionTable*, *REPTree*, 5. Conclusion and *OneR*	*OneR*, *DecisionStump*, *SMO*, and *REPTree*

## Data Availability

The data used to support the findings of this study are available from the corresponding author upon request.
